# Distribution status and influencing factors of antibiotic resistance genes in the Chaohu Lake, China

**DOI:** 10.7717/peerj.19384

**Published:** 2025-04-25

**Authors:** Yan Zhang, Guoao Ding, Yue Gao, Ying Li, Peng Zhou, Li Wu, Minghui Zhou, Jingjing Wang, Jun Tang

**Affiliations:** 1 Key Laboratory of Environmental Hormones and Reproductive Development, Anhui Province, Fuyang Normal University, Fuyang, China; 2 School of Food and Biological Engineering, Hefei Normal University, Hefei, China; 3 School of Life Sciences, Anhui Medical University, Hefei, AnHui, China

**Keywords:** Antibiotic resistance gene, Chaohu Lake, Metagenome, Antibiotic resistant bacteria

## Abstract

**Background:**

Chaohu Lake (CL) is one of the most polluted areas in China due to its high content of antibiotics. However, the distribution and influencing factors of antibiotic resistance genes (ARGs) in this lake are still controversial.

**Methods:**

To solve this problem, we used metagenomic sequencing to investigate the distribution and in-fluencing factors of ARGs in CL.

**Results:**

Our findings revealed the existence of nine kinds of ARGs, including 45 specific genes. The most abundant types were multidrug, bacitracin, polymyxin, macrolide lincosamide streptogramin, and aminoglycoside. Multiple microorganisms were undeniable ARG reservoirs, although they were not dominant species in the microbiota. Our results also showed that both the microbiota and physiochemical factors played important roles in shaping the distributions of ARGs in CL. Specifically, the levels of PO4-P (0.5927) and total phosphorus (0.4971) had a greater impact than total nitrogen (0.0515), NO_3-_N (0.0352), NO2-N (−0.1975), and NH3-N (−0.0952).

**Conclusions:**

These findings provide valuable insights into the distribution and influencing factors of ARGs in lakes.

## Introduction

In recent decades, there has been a significant increase in the emergence of antibiotic-resistant bacteria (ARB) (Mancuso et al., 2021; Sun et al., 2022). According to the World Health Organization (2022), there has been a 15% increase in human blood infections caused by antibiotic-resistant *Neisseria gonorrhoea*, *Escherichia coli*, and *Salmonella* bacteria in 2020 compared to 2017. This has attracted attention to the widespread existence of antibiotic resistance genes (ARGs) (Christaki, Marcou & Tofarides, 2020; Han et al., 2022; Weldon et al., 2022; Zhang et al., 2022; Darby et al., 2023), because environmental ARGs have been found to significantly promote antibiotic resistance (Xu et al., 2024a). Therefore, ARGs are now considered as one of the emerging pollutants in the environment (Mashima & Nakazawa, 2015; Urban-Chmiel et al., 2022; Yang & Grossart, 2024).

Due to the overuse of antibiotics and the evolution of resistors, many habitats in the environment are currently facing a threat (Zhuang et al., 2021). To effectively manage the resistance crisis, it is crucial to quantify the path and determine the driving factors and bottlenecks of environmental evolution and spread of antibiotic resistance (Larsson & Flach, 2022). The distribution of ARGs belonging to different types of antibiotics has been extensively studied. Reports from hospitals show that ARGs belonging to multidrugs, glycopeptides, and β-lactams are the most common, while reports from farms, wastewater treatment plants, water, and soil show that sulfonamides and tetracyclines are more prevalent (Zhuang et al., 2021). Xu et al. (2023) found that the absolute concentrations of ARGs are 10^2^–10^5^ copies per milliliter of water, 10^4^–10^8^ copies per gram of soil, and 10^4^–10^6^ copies per cubic meter of atmosphere. In contrast, Segawa et al. (2012) reported that ARGs are widely distributed in various glacier samples in Central Asia, North and South America, Greenland, and Africa, while there are almost no such genes in Antarctic glaciers. In addition, Yang et al. (2023) also reported high levels of quinolones, sulfonamides, and corresponding ARGs in sediments and farmland soils of fishing grounds. They further pointed out that heavy metal pollution can increase the existence of ARGs, and sheep and poultry breeding, and seawater culture are the main contributors to antibiotic resistance in the coastlines (Yang et al., 2023). These studies provide valuable data for understanding the distribution and influencing factors of ARGs.

Aquatic ecosystems are important reservoirs for ARGs (Nnadozie & Odume, 2019; Goodarzi, Asad & Mehrshad, 2022). According to Zhao et al. (2017), seawater has a significant dilution effect on the discharge of ARGs from mariculture places, and the existence of nutrients, heavy metals, and bacteria communities may directly and indirectly promote the propagation of ARGs. Cesare et al. (2023) reported that anthropogenic pollution drives the bacterial resistome in a complex freshwater ecosystem. Furthermore, aquatic environments are considered as an ideal setting for the acquisition and dissemination of antibiotic resistance as they are frequently impacted by anthropogenic activities (Marti, Variatza & Balcazar, 2014).

Chaohu Lake (CL, 31°25′–31°43′N, 117°17′–117°51′E) is the fifth-largest freshwater lake in China and divided into western (WCL) and eastern (ECL) sections (Yang et al., 2020). It is a typical shallow lake downstream of the Yangtze River (Wu et al., 2022). The landscape of the CL basin is primarily plains, and the elevation ranges from 8 to 1.49 × 10^3^ m from west to east (Wu et al., 2022). The drainage of the CL basin is 1.35 × 10^5^ km^2^ (Wu, Lai & Li, 2021). The land use types include agricultural lands (~45%), forests (~39%), built-up lands (~10%), and ponds and rivers (~6%). The average annual precipitation is 1,096 mm/year, whereas the intra-annual distribution of precipitation is uneven, with the most precipitation occurring in spring and summer. The average daily-maximum and minimum air temperature are 21.1 °C and 12.3 °C, respectively, and the daily mean air temperature is 16.7 °C (Liu et al., 2022). The CL is an important water source for industrial and agricultural production, as well as an important drinking water source (Han et al., 2022). As for as antibiotic pollution is concerned, CL is one of the most polluted areas in China. Compared with other areas, its antibiotic concentration is higher (Tang et al., 2015). Han et al. (2022) identified 75 ARG subfamilies belong to 16 ARG families through metagenomic approach and they found that multidrug and *bacA* were most frequent in the CL. Furthermore, high level of diversity and abundance of ARGs were found in the WCL with high eutrophication level, and the microbiota and MGE were the major direct factors shaping the distribution of ARGs. Moreover, Proteobacteria, Actinobacteria, Cyanobacteria, Firmicutes, and Bacteroidetes dominant the microbiota in the CL water (Han et al., 2022). However, whether the distribution and influencing factors of ARGs in CL have the same pattern in other seasons is still controversial. Therefore, the purpose of this study aimed to investigate the distribution of ARGs in another season (autumn) in CL and its influencing factors through metagenomic sequencing, and compare the results with those reported by Han et al. (2022).

## Materials and Methods

### Sample collection and processes

**Figure 1:**
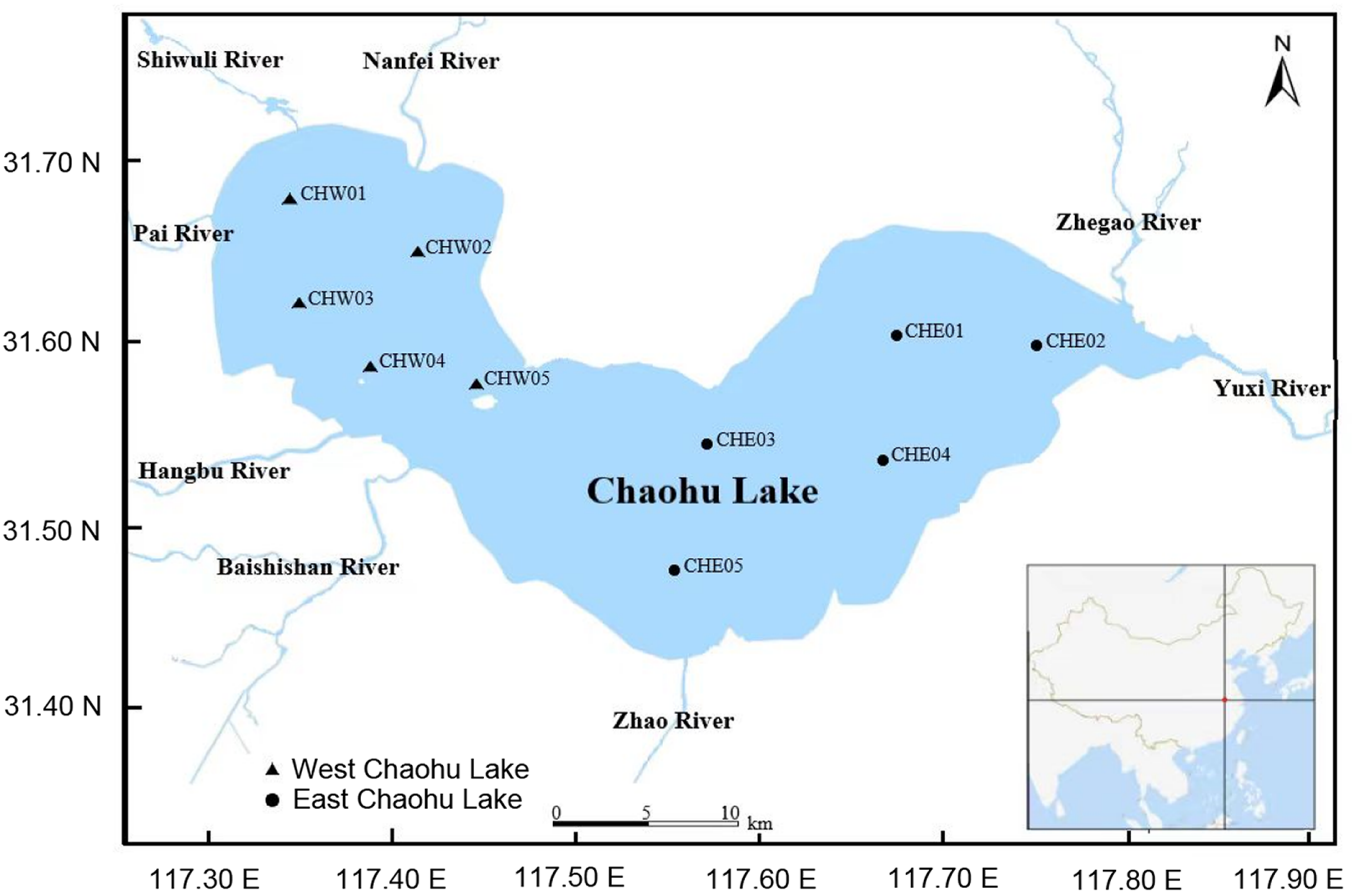
Distribution of sampling sites in Chaohu Lake.

On Oct 7, 2023, five water samples (approximately 2,000 mL for each sample) were collected from 0.5 m below the water surface by a water collector (Fig. 1 and Table S1). To ensure the representativeness of the samples, the sampling sites were relatively evenly distributed in various areas of the lake (Fig. 1 and Table S1). The water samples were temporarily stored in a box with ice and transported to the laboratory within four hours. Subsequently, the water samples were filtered using 0.22-μm filter membranes for collecting microbiota, and the filtered water was used to measure the physicochemical characterizations.

### Physicochemical analysis

The water temperature (WT), pH, dissolved oxygen (DO), and conductivity (Cond) were measured using a YSI6600-V2 multi-parameter water quality analyzer (YSI, Yellow Springs, Ohio, USA). Transparency (Trans) was measured using a Secchi disc according to the standard method (Ni et al., 2010). Chlorophyll-a content (Chl-a) was measured as previously described (Wu et al., 2022). Briefly, Chl-a was extracted from water filtered membranes using 3 mL of 100% methanol at 60 °C and their supernatant absorbances were measured on a microplate reader at 630, 647, 664, 665, and 750 nm. The levels of total phosphorus (TP), and PO_4_-P, were measured using ammonium molybdate spectrophotometric method. The level of biochemical oxygen demand after 5 days (BOD_5_) was measured using dilution and seeding method. The permanganate index (COD_Mn_) was measured using a spectrophotometric method by N,N-diethyl-p-phenylenediamine (Pang et al., 2021). The levels oftotal nitrogen (TN), NO_3_-N, NH_4_-N, and NO_2_-N were determined using alkaline potassium persulfate digestion ultraviolet spectrophotometric method, ultraviolet spectrophotometry, Nessler’s reagent spectrophotometry, and spectrophotometric method, respectively (Huang, 2000). The physicochemical indices of each sample were measured three times repeatedly and averaged as the result.

### DNA extraction and metagenomic sequencing

The metagenomic DNA was extracted from the filter membrane of each water sample using an E.Z.N.A. water DNA kit (OMEGA Bio-tek, Houston, TX, USA) as Pearce et al. (2009). Shotgun metagenomic sequencing was then performed according to the protocol outlined by Han et al. (2022). Trimmomatic v0.32 was used to filter out low-quality reads, as described in Han et al. (2022). Briefly, the metagenomic DNA of each sample was detected by 1.2% agarose gel electrophoresis and Nanodrop (Thermo Fisher Scientific, Waltham, MA, United States), and then randomly fragmented of the desired size by Covaris S/E210 (Covaris, Woburn, MA, USA) and required-length DNA fragments (approximate 300 bp) were purified using 1.8% agarose gel electrophorisis. Subsequently, the sequencing adapters were ligated to the DNA fragments and prepared for clustering and library sequencing. The library was PE150 sequenced on a Xten platform (Illumina, Madison, WI, USA). Trimmomatic v0.32 was used to filter out low-quality reads, as described in Han et al. (2022). Next, the high-quality reads from each sample were *de novo* assembled using MEGAHIT, following the method described by Han et al. (2022). The contigs with a length longer than 500 bp were maintained and metagenomics binning was conducted using Metabat2 (Kang et al., 2019) with default parameters and binning quality was checked using CheckM v1.2.3 (Parks et al., 2015) with default parameters. Taxonomically annotated using MetaPhlAn4 with mpa vOct22_CHOCOPh1AnSGB_202212 database and default parameters (Blanco-Míguez et al., 2023), and genes and their locations were predicted using MetaProdigal with default parameters (Hyatt et al., 2012) and ARGs were identified from the predicted genes using the ARGs-OAP v2.0 with an expanded SARG database version 2.0 (Yin et al., 2018). To obtain the relationship between ARGs and mobile genetic elements (MGEs), the protein sequences on ARG-carrying contigs were extracted and annotated them against the National Center for Biotechnology Information non-redundant protein database using BLASTP according to previously described (Han et al., 2022). Then the predicted gene annotations and open reading frame arrangements on ARG-MGE contigs were obtained and visualized with geneplot function in R. Plasmid and chromosomal sequences from ARG-MGE contigs were predicted using PlasFlow 1.1 (Smillie et al., 2010).

### Data analysis

The relative abundance of each ARG in the samples was calculated as previously described by Han et al. (2022) to remove the effects of sequencing depth and length of ARG on subsequent results. Wilcoxon rank sum test was performed using R to determine any differences between WCL and ECL in physicochemical parameters and α-diversity indices. Box and violin diagrams were created using the R ggpubr package. Linear discriminant analysis (LDA) was performed using the R MASS and ggplot2 packages. For co-occurrence network analysis with Spearman correlations, isometric log-ratio transformation of data was conducted using R with mkin package. Co-occurrence networks with Spearman correlations were built using R with the igraph, psych, and Hmisc packages, and then visualized using Gephi 0.9.2 (https://gephi.org/) (Bastian, Heymann & Jacomy, 2009). The correlations with Spearman correlation coefficient more than 0.7 and *P* value < 0.05 were significant. Partial least squares path modeling (PLS-PM) analysis was performed using the R plspm package (https://www.gastonsanchez.com/PLS_Path_Modeling_with_R.pdf). The bar plots and other charts were drawn using the R ggplot2 package.

## Results

### Physicochemical parameter

**Figure 2:**
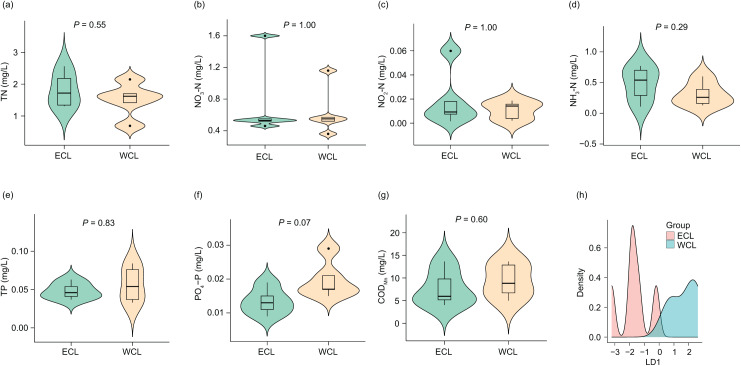
Differences in water physicochemical parameters between Western and Eastern Chaohu Lakes. (A) Total nitrogen (TN) concentration; (B) NO_3_-N concentration; (C) NO_2_-N concentration; (D) NH_4_-N concentration; (E) total phosphorous (TP) concentration; (F) PO_4_-P; (G) chemical oxygen demand (COD_Mn_); (H) density distribution of LD1 values calculated based on physicochemical factors of water samples between Western and Eastern Chaohu Lakes.

Considering that the physicochemical parameters of water affect the composition and abundance of ARGs through affecting water microbiota (Zhou et al., 2017; Rajasekar et al., 2023), we first analyzed the physicochemical parameters of water samples. The concentrations of TN (1.67 ± 0.168 mg/L), NO_3_-N (0.68 ± 0.122 mg/L), NO_2_-N (0.02 ± 0.005 mg/L), NH_3_-N (0.40 ± 0.077 mg/L), TP (0.05 ± 0.005 mg/L), PO_4_-P (0.02 ± 0.002 mg/L), and COD_Mn_ (8.56 ± 1.185 mg/L) in the samples were lower than those reported in a previous study (Han et al., 2022). However, although previous research has shown that the nitrogen and phosphorous levels in the WCL are significantly higher than those in the ECL (Han et al., 2022), our results did not find a significant difference in these physicochemical parameters between the two lakes (*P* > 0.05; Fig. 2). In addition, our LDA results showed that the discrimination formula was:

$\eqalign{{\rm LD}1 =\ & -3.38 \times {\rm TN} - 55.50 \times {\rm TP}-0.43 \times ({\rm N}{{\rm H}_3}-{\rm N}) + 8.46 \times \left( {{\rm N}{{\rm O}_3}-{\rm N}} \right)-171.64\\ & \times  \left( {{\rm N}{{\rm O}_2}-{\rm N}} \right) + 305.91 \times \left( {{\rm P}{{\rm O}_4}-{\rm P}} \right) + 0.13 \times {\rm CO}{{\rm D}_{{\rm Mn}}.}}$

This formula allowed for complete and accurate distinction between the ECL and WCL samples (Fig. 2H and Fig. S1).

### Characteristics and profile of ARGs

**Figure 3:**
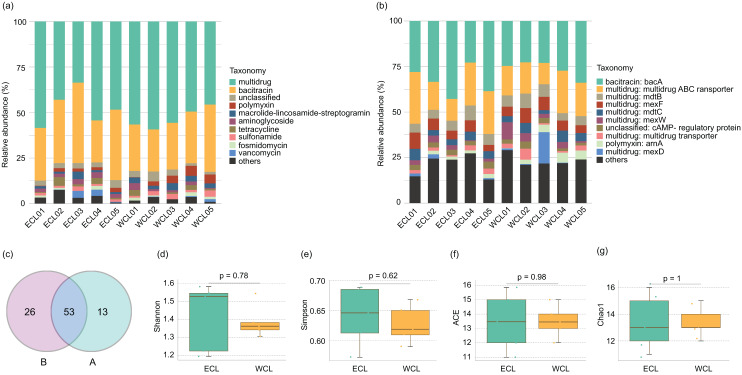
Composition of antibiotic resistance genes (ARGs) in the Chaohu Lake. (A) Relative abundance of different ARG types in each sample; (B) relative abundance of different ARG genes in each sample; (C) shared ARG genes between the Western and Eastern Chaohu Lake samples; (D) Shannon index; (E) Simpson index; (F) ACE index; (G) Chao1 index. The ARGs were identified from the predicted genes using the ARGs-OAP v2.0 with an expanded SARG database version 2.0 (Yin et al., 2018).

Shotgun metagenomic sequencing has been widely used to monitor the distribution of ARGs (Hendriksen et al., 2019; Filipic et al., 2020; Manoharan et al., 2021; Díaz-Torres et al., 2024; Xiao et al., 2025). To analyze the composition of ARGs in CL, a total of 208.44 Gb high-quality reads were obtained from the shotgun metagenomic sequencing in the ten water samples (Table S1). An average of 143.4 metagenome-assembled genomes were obtained from metagenome samples through binning (Table S1). Except for unclassified ARGs, nine families of ARGs were detected in CL, which contained 45 genes (Fig. 3A and Table S2). The five most abundant types of ARGs in CL were multidrug, bacitracin, polymyxin, macrolide lincosamide streptogramin (MLS), and aminoglycoside (Fig. 3A). The most prevalent ARG was *bacA* (29.74 ± 2.25%), followed by the multidrug ABC transporter gene (18.96 ± 1.75%), *mexF* (6.01 ± 0.55%), and *mdtB* (5.94 ± 0.44%; Fig. 3B and Table S2). Geographically, 79 ARGs were found in the WCL and 66 ARGs were found in the ECL. In addition, 53 ARGs were shared between WCL and ECL (Fig. 3C). The diversity indices (Shannon, Simpson, ACE, and Chao1 indices) of ARGs did not show significant difference between WCL and ECL (Figs. 3D–3G).

**Figure 4:**
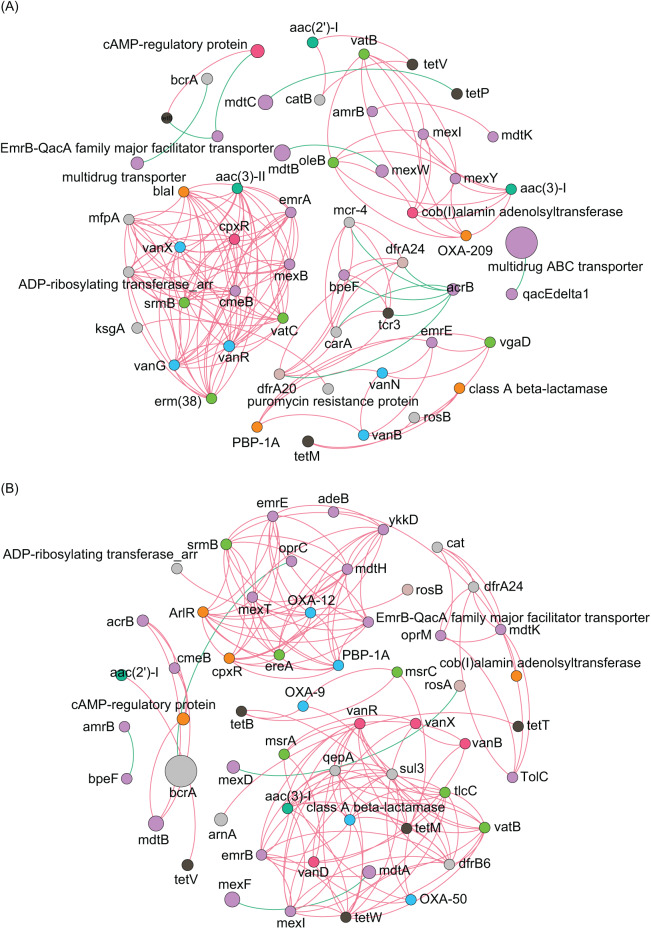
Spearman co-occurrence networks among antibiotic resistance genes (ARGs) subtypes. (A) Network among ARG subtypes in the Eastern Chaohu Lake samples; (B) network among ARG subtypes in the Western Chaohu Lake samples. Spearman correlation coefficient > 0.7 and *P* < 0.01 were considered as effective correlation.

Co-occurrence network analysis of ARGs showed that the network included 54 nodes and 158 edges, and the average clustering coefficient was 1.000 in the ECL, and in the WCL, there were 56 nodes and 150 edges, and the average clustering coefficient was 1.000 (Fig. 4). These findings demonstrated a similar co-occurrence of ARGs in the ECL and WCL.

### Characterization of microbiota and correlation between microbiota and ARGs

**Figure 5:**
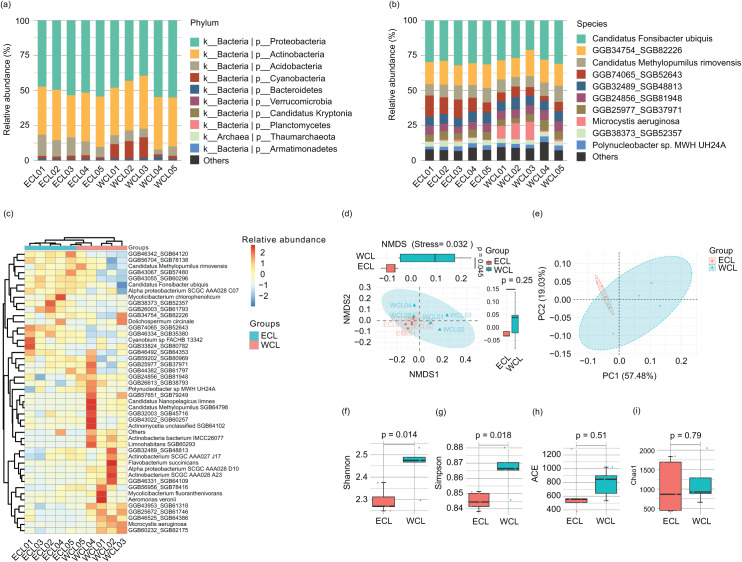
Microbiota compositions of Chaohu Lake water. (A) Composition of main phyla in the microbiota; (B) Composition of main species in the microbiota; (C) heatmap profile shows the clustering results of samples; (D) non-metric multidimensional scaling (NMDS) profile; (E) principal coordinate analysis (PCoA) profile; (F) Shannon index; (G) Simpson index; (H) ACE index; (I) Chao1 index.

Because the water microbiota is the reservoir of ARGs (Nnadozie & Odume, 2019; Goodarzi, Asad & Mehrshad, 2022), the changes of microbiota structure would affect the composition of ARGs. Therefore, we analyzed the microbiota structure in the CL water. A total of 11 bacterial phyla (Proteobacteria, Actinobacteria, Acidobacteria, Cyanobacteria, Bacteroidetes, Candidatus_Kryptonia, Verrucomicrobia, Planctomycetes, Nitrospirae, Armatimonadetes, and Candidatus_Saccharibacteria) and 1 archaeal phylum (Thaumarchaeota) were detected in the water samples. Proteobacteria (39.50–55.02%) and Actinobacteria (30.14–37.79%) were the most abundant phyla (Fig. 5A and Table S3), consistent with other natural waters (Dang et al., 2020; Wang et al., 2020, Zhang et al., 2021). At the species level, 194 species were detected in the water samples. The dominant species were *Candidatus Fonsibacter ubiquis* (28.80 ± 1.00%), GGB34754_SGB82226 (15.71 ± 0.51%), GGB74065_SGB52643 (8.77 ± 1.15%), *Candidatus Methylopumilus rimovensis* (9.21 ± 0.44%), GGB24856_SGB81948 (6.07 ± 0.23%), GGB32489_SGB48813 (7.05 ± 0.21%), GGB25977_SGB37971 (5.35 ± 0.34%), GGB38373_SGB52357 (3.50 ± 0.12%), *Polynucleobacter* sp. MWH_UH24A (3.00 ± 0.19%), *Cyanobium* sp. FACHB_13342 (0.91 ± 0.08%), GGB43067_SGB57480 (1.37 ± 0.12%), actinobacterium SCGC_AAA028_A23 (1.08 ± 0.14%), GGB43022_SGB60257 (0.61 ± 0.08%), *Microcystis aeruginosa* (3.88 ± 1.63%), *Dolichospermum circinale* (0.27 ± 0.12%), and *Mycolicibacterium fluoranthenivorans* (0.23 ± 0.16%) (Fig. 5B and Table S3). Cluster analysis based on major species showed that the samples were clustered according to their geographical locations (WCL and ECL) (Fig. 5C). Non-metric multidimensional scaling (NMDS) and principal coordinate analysis (PCoA) also indicated that the samples were distinguished by geographical positions (Figs. 5D and 5E). In addition, the Shannon and Simpson indices of WCL were significantly higher than those of ECL (Figs. 5F and 5G), while the ACE and Chao1 indices did not show significant differences between WCL and ECL (Figs. 5H and 5I).

**Figure 6:**
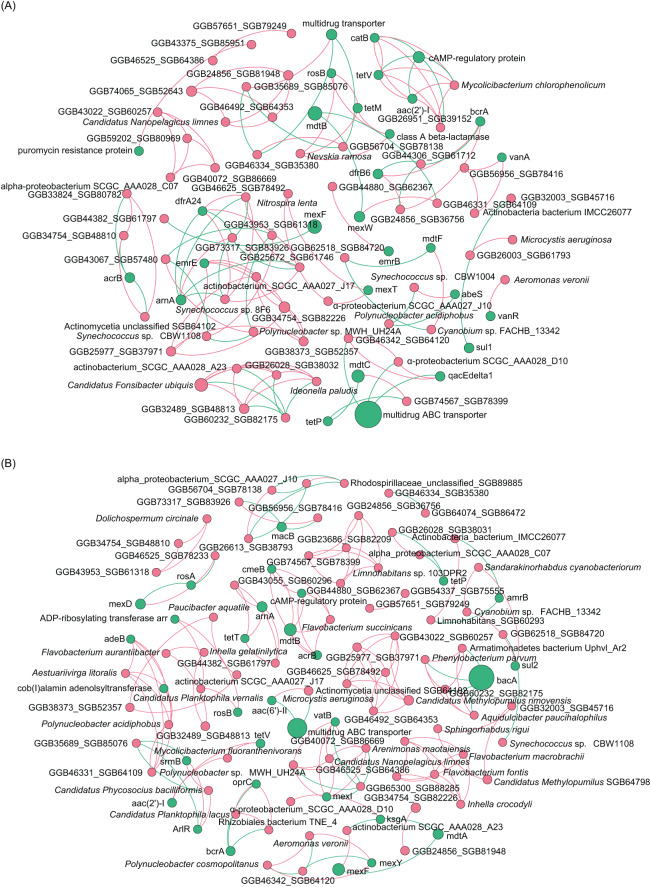
Spearman co-occurrence network analysis of the correlation between antibiotic resistance gene (ARG) subtypes and microorganisms in the (A) Eastern and (B) Western Chaohu Lake samples. Spearman correlation coefficient > 0.7 and *P* < 0.01 were considered as effective correlation.

To analyze the co-occurrence relationship between ARGs and bacterial species in the aquatic microbiota, we conducted co-occurrence network analysis with Spearman correlation. Our results revealed that in the ECL, *rosB*, *tetM*, and class *A β-lactamase* were significantly positively correlated with *Nevskia ramosa*. *catB*, *tetV*, and *aac(2’)-I* were significantly positively correlated with *Mycolicibacteirum chlorophenolicum*, and GGB26951_SGB39152, whereas they were significantly negatively correlated with cAMP-regulatory protein. *vanR* was significantly positively correlated with *Aeromonas veronii. abeS* was significantly positively correlated with *Synechococcus* sp. CBW1004, whereas was significantly negatively correlated with *Polynucleobacter acidiphobus*. *mexT* was significantly positively correlated with *Nitrospira lenta* and α-proteobacteirum SCGC_AAA027_J10. *dfrA24* was significantly positively correlated with *Synechococcus* sp. 8F6, were as *arnA* was significantly negatively correlated with the species (Fig. 6A). In contrast, in the WCL, *adeB* and *cob(I)alamin adenolsyltransferase* were significantly positively correlated with *Aestuariivirga litoralis*, *Polynucleobacter acidiphobus*, and two unidentified species *rosB* and *ADP-ribosylating transferase arr* were significantly correlated with *Candidatus Planktophila vernalis. tetV* and *aac(2’)-I* were significantly positively correlated with *Mycolicibacterium fluoranthenivorans*. *mexY* was significantly positively correlated with *Aeromonas veronii*. *mexI* and *vatB* were significantly positively correlated with *Arenimonas maotaiensis* and *Candidatus Nanopelagicus limnes*. *bacA* was significantly negatively correlated with Armatimonadetes bacteirum Uphvl_Ar2, *Phenylobacterium parvumm*, and *Aquidulcibacter paucihalophilus*. *cmeB*, *mdtB*, *acrB*, and *cAMP-regulatory protein* were signficantly positively correlated with *Flavobacteirum succinicans*. *sul2* was significantly positively correalted with *Cyanobium* sp. FACHB_13342 and *Sandarakinorhabdus cyanobacteriorum* (Fig. 6B). These results indicated that a variety of microorganisms were undeniable ARG reservoirs, although they were not dominant species in the microbiota.

### Distribution pattern of ARGs in mobile genetic elements (MGEs)

**Figure 7:**
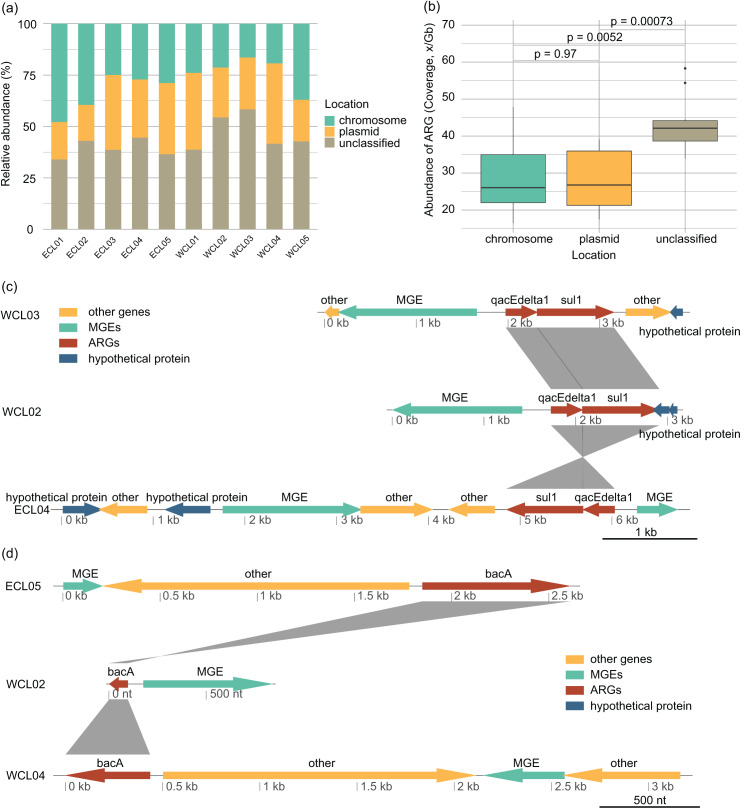
Source-tracking of assembled contigs carrying antibiotic resistance gene (ARG) or ARG-MGE and typical arrangements of ARGs and MGEs in assembled contigs. (A) Proportions of ARGs located on plasmid, chromosomal, and unclassified sequences; (B) proportions of ARGs co-occurring with MGEs located on plasmid, chromosomal, and unclassified sequences; (C) different arrangement of *sul1* and *qacEdelta* gene cluster with other ARGs and MGEs; (D) different arrangement of *bacA* gene with MGEs.

The distribution of ARGs in chromosomes and plasmids and their relationships with MGEs affect their horizontal transfer ability (Castañeda-Barba, Top & Stalder, 2024). In all the samples, the proportion of ARGs located on plasmids (28.13 ± 2.61% of the total ARG abundance) was roughly equivalent to that on chromosomes (28.64 ± 3.13%) (Wilcoxon rank sum exact test, W = 51, *P* = 0.97; Figs. 7A and 7B). The changes in the environment altered the way of ARGs combining with MGEs, with *sul1-qacEdelta1* being one of the most common combinations (Fig. 7C). However, *bacA* was typically found to be separated from MGEs by other genes (Fig. 7D).

### Factors affecting the profiles of ARGs

**Figure 8:**
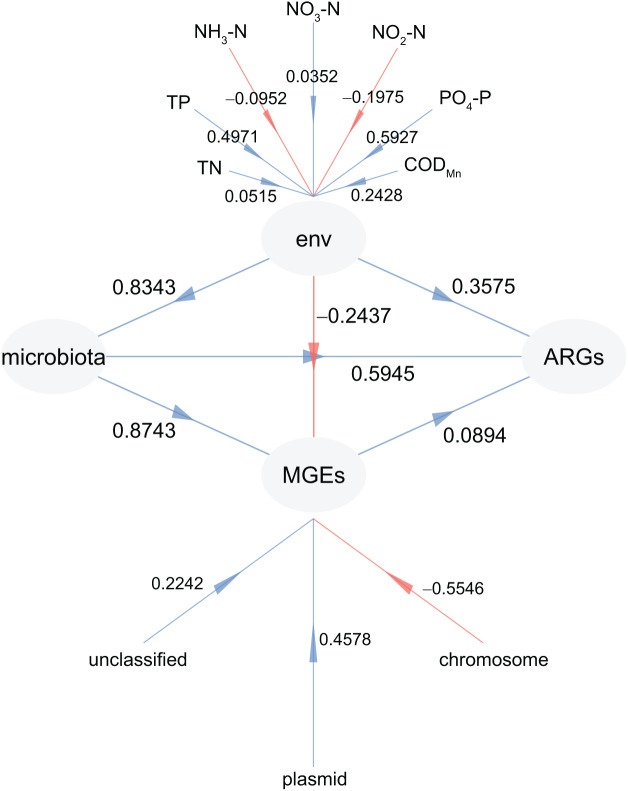
Partial least squares path modeling (PLS-PM) results reveal the relationship between physiochemical factors (env), MGEs, microbiota, and ARGs.

To investigate the factors driving the distribution of ARGs in CL, PLS-PM analysis was adopted. This allowed us to examine the impact of physiochemical factors, MGEs, and microbiota on the profiles of ARGs (Fig. 8). Our findings revealed that microbiota had the greatest influence on both ARGs (0.5945) and MGEs (0.8743), followed by physiochemical factors (0.3575 for ARGs and 0.5945 for MGEs) (Fig. 8). In addition, the weights of PO_4_-P (0.5927) and TP (0.4971) in the physiochemical factors were higher than those of TN (0.0515), NO_3_-N (0.0352), NO_2_-N (−0.1975), and NH_3_-N (−0.0952; Fig. 8). Furthermore, the weight of plasmid in the MGEs was significantly higher than that of the chromosome (−0.5546; Fig. 8). These results confirm that the microbiota and physiochemical factors are the primary drivers of ARG distribution in CL, with plasmids being the main location for MGEs compared to the chromosome.

**Figure 9:**
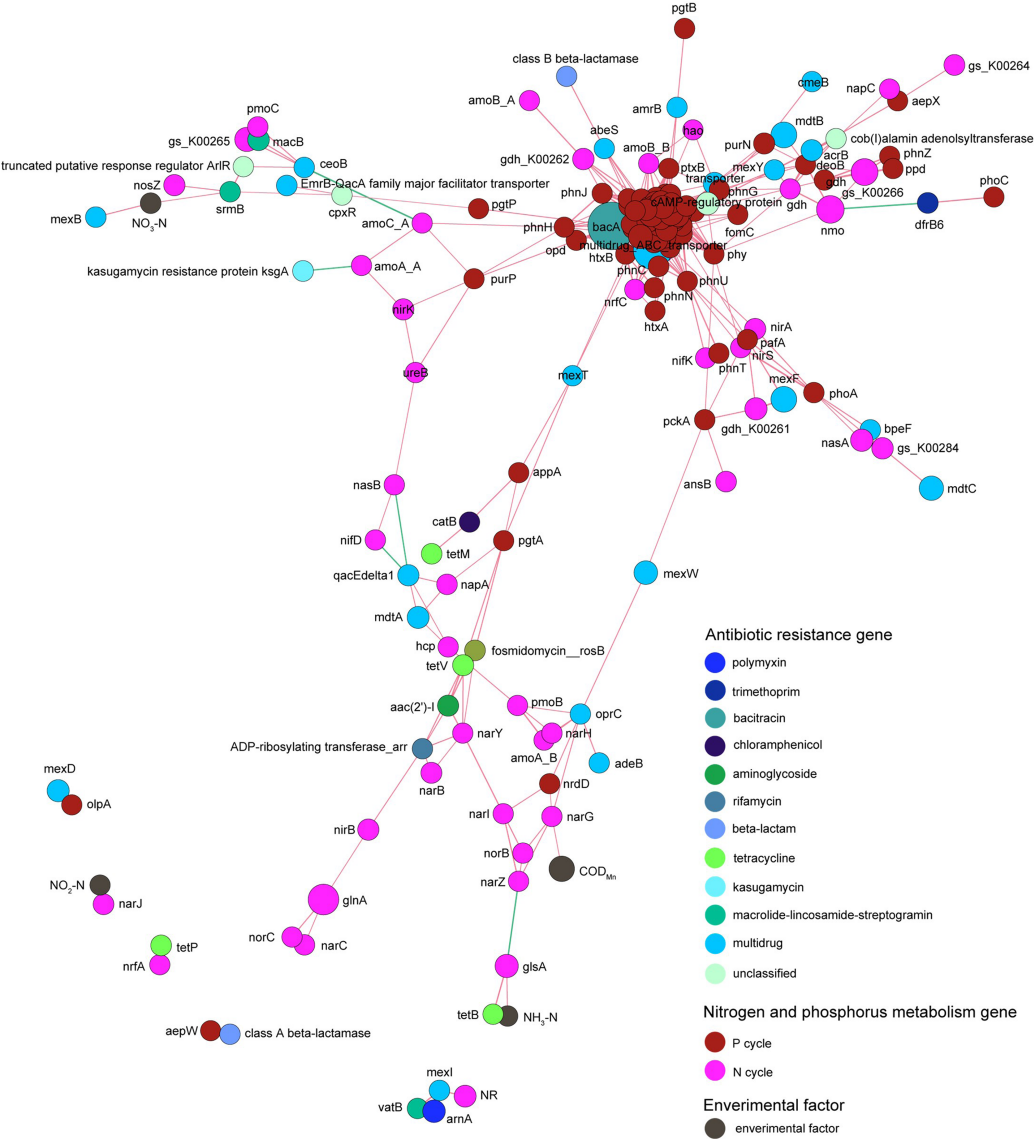
Spearman co-occurrence network shows the correlation between the antibiotic resistance genes, nitrogen and phosphorus metabolism genes, and environmental factors. Spearman correlation coefficient > 0.7 and *P* < 0.01 were considered as effective correlation.

Co-occurrence network analysis revealed that the correlation between genes related to nitrogen metabolism and antibiotic resistance genes was obviously more complicated than that between genes related to phosphorus metabolism and antibiotic resistance genes. Genes related to nitrogen and phosphorus metabolism did not exhibit absolute correlation with the contents of nitrogen and phosphorus contents in the pond water (Fig. 9).

## Discussion

Excessive use of antibiotics and discharge of wastewater into lakes are the main contributing factors to the high prevalence of ARGs in lake microbiota (Yang et al., 2018). In a previous study, Han et al. (2022) detected 16 ARG types with 75 subtypes in the CL water collected on 17 April 2018. Zhang et al. (2023) found a significant positive correlation between antibiotics and ARGs in sediments of Wanfeng Lake. The positive associations between ARGs and antibiotics were also reported in another lake (Xu et al., 2024b). Nanfei River and Shiwuli River account for 66% of the total amount of antibiotics in all eight rivers, and they are probably the main source of antibiotics in CL (Tang et al., 2015), which is considered as the main reason caused the ARG differences between WCL and ECL (Han et al., 2022). However, our results did not show a significant difference in ARGs between the WCL and ECL, despite their microbiota structures are distinct.

The high prevalence rate of antibiotic resistance is associated to the increase of regional ambient temperature (MacFadden et al., 2018; McGough et al., 2020; Li et al., 2023). This may be due to temperature improves plasmid-mediated dissemination of antibiotic resistance (Pallares-Vega et al., 2021). Another factor that affects ARG content is the nutritional status. Low nutrient media led to a significant reduction in the transconjugants of an IncP-1 (pKJK5) plasmid to three natural *E. coli* strains carrying extended-spectrum β-lactamases (Pallares-Vega et al., 2021). Yang et al. (2020) reported that the total annual nutrient input into the WCL was approximately 12 times higher than that put into the ECL. A previous study has shown that the nitrogen source was the main physicochemical factor affecting ARG occurrence and distribution compared with phosphorus source in CL. However, our results indicated that the weights of PO_4_-P (0.5927) and TP (0.4971) in the physicochemical factors were higher than TN (0.0515), NO_3_-N (0.0352), NO_2_-N (−0.1975), and NH_3_-N (−0.0952). This suggests that the phosphorus source is the main physicochemical factor affecting the occurrence and distribution of ARGs in CL. As an important part of lake nutrient and the close relationship between eutrophication, the relationship between phosphorus concentration in sediments and suspended particulate matter and occurrence of algal bloom has been widely concerned (Li et al., 2024). Simultaneously, the role of microorganisms in phosphorus circulation in activated sludge and sediment has also been widely studied (Zheng et al., 2019; Yuan et al., 2024; Liang et al., 2025). Although studies have shown that nitrogen has a greater influence on the microbiota structure in natural water than phosphorus (Liu et al., 2020; Fan et al., 2022), this is probably because the variation of nitrogen concentration in water is greater than that of phosphorus, which makes it easier to detect the significant correlation with microbiota. Our results suggested that as the container of ARGs, water microbiota may be more restricted by phosphorus than nitrogen, although this inference needs further experimental verification. Therefore, it is necessary to further explore the influence of nutritional factors on the occurrence and distribution of ARGs.

Bai et al. (2022) reported that bacitracin, multidrug, and sulfonamides resistance gene were with the highest abundance in overlying water and sediment at typical area of Taihu Lake. The prevalence of multidrug resistance genes in prokaryotes may be due to the selective pressure of multiple antibiotics and the widespread existence of multidrug resistance efflux pumps. These genes have been found in a variety of aquatic environments, such as rivers, lakes, and aquaculture ponds (Guo et al., 2018; Ju et al., 2018; Yang et al., 2019; Chen et al., 2020). Our study also revealed that multidrug resistance was the most common type of ARG in CL, which is consistent with a previous study (Han et al., 2022).

Hospitals are generally regarded as an important repository and source of ARGs (Chng et al., 2020). However, increasing attention is being turned toward environmental pathways that potentially contribute to ARG dissemination outside the clinical realm recently (Li et al., 2020). Although hospital sewage is considered to be an important source of antibiotics and ARGs, Yuan et al. (2022) found that animal manure had higher levels of detected ARGs and MGEs than hospital effluent, with the exception of the *sul1* gene. The study also identified potential hosts of ARGs, such as *Jeotgalibaca*, *Atopostipes*, and *Corynebacterium_1*, which are more prevalent in animal manure than in hospital sewage. This indicates that animal manure may have a higher potential for ARG transfer compared to hospital sources. Zhang et al. (2022) analyzed 4,572 metagenomic samples collected from air, aquatic, terrestrial, engineered, humans and other hosts to illustrate the global patterns of ARG distribution, and they identified a 2,561 ARGs that conferred resistance to 24 drug classes of antibiotics. Furthermore, they found the human-associated habitats, including the digestive system and skin, had the highest abundances of ARGs. Built environments, mainly including urban subways, also had considerable abundance of ARGs, confirming these as hotspots of ARGs. In addition, the waste discharge from veterinary antibiotic manufacturing sites also brings a risk for horizontal transfer of ARGs (Miao et al., 2021). Díaz-Torres et al. (2024) investigated the occurrence and distribution of ARGs in Lake Cajititlán, and they detected 475 ARG genes in 22 bacterial genera, *Pseudomonas* (144 ARG genes), *Stenotrophomonas* (88 ARG genes), *Mycobacterium* (54 ARG genes), and *Rhodococcus* (27 ARG genes) displaying the highest frequencies of ARGs. *Pseudomonas aeruginosa* and *Stenotrophomonas maltophilia* showed the highest number of ARGs. In another study, Han et al. (2022) explored the location of ARGs in CL to gain further insights into their distribution pattern. The results showed that the prevalence of ARGs in rural sediments was promoted by both horizontal and vertical gene transfer, while their spread in surface waters and soils was facilitated by biogenic elements and HGT, respectively (Cheng, Tang & Liu, 2020). In addition, the abundance of MGEs was significantly related to the abundance of ARGs, which indicates that ARGs may be horizontally transferred to other bacteria and pathogens in rural environments (Cheng, Tang & Liu, 2020). Our results also showed that the change of environment can affect the way that ARGs combine with MGEs, among which *sul1-qacEdelta1* is the most representative.

In our study, we discovered that the most prevalent ARG was *bacA* (29.74 ± 2.25%), followed by the multidrug ABC transporter gene (18.96 ± 1.75%), *mexF* (6.01 ± 0.55%), and *mdtB* (5.94 ± 0.44%; Fig. 3B and Table S2). The main mechanisms of antimicrobial resistances include lack of antibiotic targets, enzymatic inactivation through disintegration or chemical modification, reduced intracellular accumulation through decreased influx or increased efflux, and modifications at the cellular target sites such as mutational changes, chemical modification, protection, or even replacement of the target sites (Van Duijkeren et al., 2017). For instance, cell wall-free bacteria, such as *Mycoplasma* spp., are resistant to β-lactam antibiotics and glycopeptides (Van Duijkeren et al., 2017). In *Escherichia coli*, *bacA*, which codes for an undecaprenyl pyrophosphate phosphatase, is responsible for bacitracin resistance (El Ghachi et al., 2004). An ABC transporter that mediates bacitracin resistance was identified on a conjugative plasmid in *Enterococcus faecalis* (Manson et al., 2004). Avilamycin resistance in the producer organism *Streptomyces viridochromogenes* Tü57 is based on the activity of an ABC transporter and two rRNA methyltransferases (Weitnauer et al., 2001). MexAB-OprM and MexEF-OprN are *Pseudomonas aeruginosa* efflux pumps involved in the development of antibiotic resistance (Horna et al., 2018). The heterotimeric MdtB_2_C complex of *E. coli* is the functional form of the MdtB-MdtC RND family transporter, as supported by genetic studies showing that MdtB and MdtC are interdependent for efflux activity (Baranova & Nikaido, 2002; Kim, Nagore & Nikaido, 2010).

It should be noted that there are still some limitations in this study. Firstly, although we found that there were seasonal differences in the distribution of ARGs in CL by comparing with the results of Han et al. (2022), the seasonal distribution of ARGs in CL needs to be sampled and analyzed more frequently, such as metagenome sequencing and ARG analysis though sampling regularly every month. Secondly, although CL is the fifth largest lake in China and occupies an important position in industrial and agricultural production in the lower reaches of the Yangtze River, it is still unclear whether the distribution of ARGs in CL is consistent with other lakes in the middle and lower reaches of the Yangtze River, or whether there are obvious regional differences. Cross-lake and cross-regional investigations of ARGs are of great value for analyzing the source and diffusion patterns of ARGs. Thirdly, although we found that physiochemical factors, especially phosphorus, were important factors affecting the distribution of ARGs, we have not clarified the mechanisms of physiochemical factors affecting the distribution of ARGs. Revealing the mechanism of influence of physiochemical factors on ARG distribution can help us to establish the technology of regulating ARG distribution through physiochemical factors.

## Conclusions

ARGs of multidrug, bacitracin, polymyxin, macrolide lincosamide streptogramin (MLS), and aminoglycoside were the five most abundant ARG types in CL. Multiple microorganisms were undeniable ARG reservoirs, although they were not dominant species in the microbiota. Microbiota and physiochemical factors were the main factors shaping the distributions of ARGs in CL. Comparing with previously studies, there were seasonal differences in the distribution of ARGs in CL. These results had important reference significance for us to gain a deeper understanding of the distribution and influencing factors of ARGs in lakes, in particular, seasonal differences in the distribution of ARGs mean that measures to control ARGs may need to be changed according to seasonal differences. The lack of sampling time in this study (sampling only at a single time) and the limitation of the number of lake (only one lake) lead to the results not being widely representative, so the distribution of ARGs in the follow-up needs to be studied more widely across seasons and lakes. The factors directly affecting the distribution of ARGs also need to be further revealed to provide more direct practical guidance for controlling the diffusion of ARGs.

## Supplemental Information

10.7717/peerj.19384/supp-1 Supplemental Information 1 LDA results.

10.7717/peerj.19384/supp-2 Supplemental Information 2 R script using in this study.

10.7717/peerj.19384/supp-3 Supplemental Information 3 Basic data of each sample and sequencing data obtained by shotgun metagenomic sequencing after quality control.

10.7717/peerj.19384/supp-4 Supplemental Information 4 Relative abundances of anbitiobic resistance genes in the Chaohu Lake.

10.7717/peerj.19384/supp-5 Supplemental Information 5 Relative abundances of phyla in the Chaohu water microbiota.

10.7717/peerj.19384/supp-6 Supplemental Information 6 Dominant species in the Chaohu Lake water.

## Additional Information and Declarations

### Data Availability

The following information was supplied regarding data availability:

The data presented in this study are openly available at NCBI Sequence Read Archive: PRJNA1109553.
