# Supplementary figures and images for: Distribution status and influencing factors of antibiotic resistance genes in the Chaohu Lake, China
Source: PeerJ. 2025 Apr 25;13:e19384. doi: 10.7717/peerj.19384 (PMC12036580; doi:10.7717/peerj.19384)

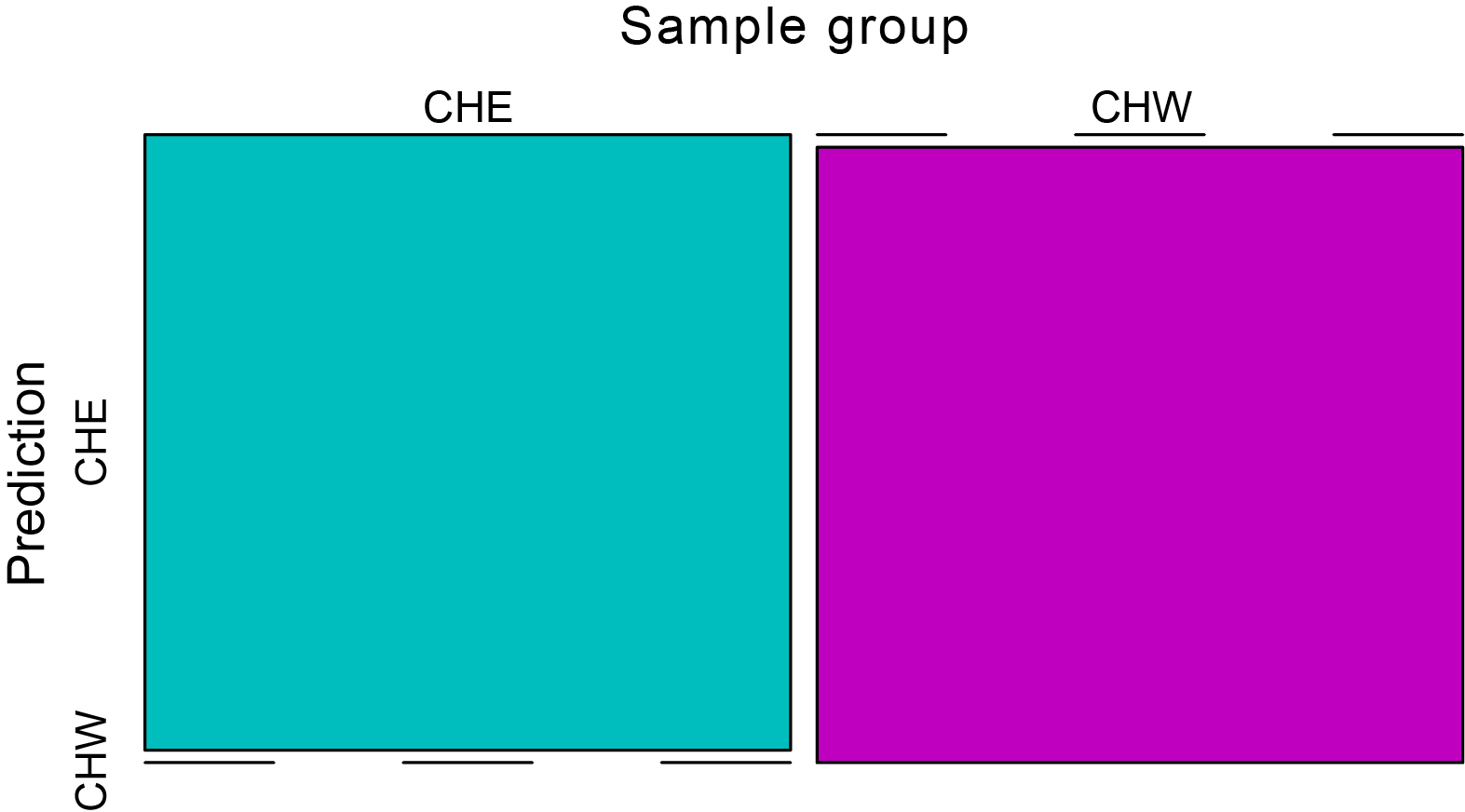

Supplement: Supplemental Information 1 [file peerj-13-19384-s001.jpg]
