# Supplementary material for: Distribution status and influencing factors of antibiotic resistance genes in the Chaohu Lake, China
Source: PeerJ. 2025 Apr 25;13:e19384. doi: 10.7717/peerj.19384 (PMC12036580; doi:10.7717/peerj.19384)
Supplement: Supplemental Information 2 [file peerj-13-19384-s002.docx]

Supplementary Materials-Scripts

# Co-occurrence network analysis scripts of R

# R version 4.2.3

library(mkin)

library(igraph)

library(psych)

library(Hmisc)

E.antibiotic<-read.csv("C:/R/E_antibioti-gene.csv",header=T,row.names=1)

E.antibiotic<-replace(E.antibiotic,E.antibiotic<0.000001, 0.000001)

E.antibiotic$CHE01<-invilr(ilr(E.antibiotic$CHE01))

E.antibiotic$CHE02<-invilr(ilr(E.antibiotic$CHE02))

E.antibiotic$CHE03<-invilr(ilr(E.antibiotic$CHE03))

E.antibiotic$CHE04<-invilr(ilr(E.antibiotic$CHE04))

E.antibiotic$CHE05<-invilr(ilr(E.antibiotic$CHE05))

E.antibiotic<-t(E.antibiotic)

occor = corr.test(E.antibiotic,use="pairwise",method="spearman",adjust="fdr",alpha=.05)

occor.r = occor$r

occor.p = occor$p

occor.r[occor.p>0.01|abs(occor.r)<0.7] = 0

igraph = graph_from_adjacency_matrix (occor.r, mode="undirected", weighted=TRUE, diag=FALSE)

Igraph

bad.vs = V(igraph)[degree(igraph) == 0]

igraph = delete.vertices(igraph, bad.vs)

igraph

igraph.weight = E(igraph)$weight

write_graph(igraph, "C:/R/E_antibioti_gene.graphml","graphml")

W.antibiotic<-read.csv("C:/R/W_antibioti-gene.csv",header=T,row.names=1)

W.antibiotic<-replace(W.antibiotic,W.antibiotic<0.000001, 0.000001)

W.antibiotic$CHW01<-invilr(ilr(W.antibiotic$CHW01))

W.antibiotic$CHW02<-invilr(ilr(W.antibiotic$CHW02))

W.antibiotic$CHW03<-invilr(ilr(W.antibiotic$CHW03))

W.antibiotic$CHW04<-invilr(ilr(W.antibiotic$CHW04))

W.antibiotic$CHW05<-invilr(ilr(W.antibiotic$CHW05))

W.antibiotic<-t(W.antibiotic)

occor = corr.test(W.antibiotic,use="pairwise",method="spearman",adjust="fdr",alpha=.05)

occor.r = occor$r

occor.p = occor$p

occor.r[occor.p>0.01|abs(occor.r)<0.7] = 0

igraph = graph_from_adjacency_matrix (occor.r, mode="undirected", weighted=TRUE, diag=FALSE)

igraph

bad.vs = V(igraph)[degree(igraph) == 0]

igraph = delete.vertices(igraph, bad.vs)

igraph

write_graph(igraph, "C:/R/W_antibioti_gene.graphml","graphml")

# LDA script

library(ggplot2)

library(MASS)

mydata<-read.csv("C:/R/env.csv",header=T,row.names=1)

mydata$group<-as.factor(mydata$group)

attach(mydata)

lakeld <- lda(group ~ TN + TP + NH3.N + NO3.N + NO2.N + PO4 + CODMn, data = mydata)

summary(lakeld)

lakeld

lakePredict <- predict(lakeld)

newGroup <- lakePredict$class

cbind(mydata$group, lakePredict$x, newGroup)

tab <- table(mydata$group , newGroup)

erro <- 1-sum(diag(prop.table(tab)))

plot(tab)

LDAresults<-read.csv("C:/R/LDA_DATA.csv",header=T,row.names=1)

qplot(x=LD1,data = subset(LDAresults,!is.na(group)),geom = 'freqpoly')

p<-ggplot(LDAresults, aes(x = LD1))

p + geom_density(aes(color = group))

LDAresults$group<-as.factor(LDAresults$group)

p + geom_density(aes(fill = group), alpha=0.4)+theme_classic()
